# Supplementary figures and images for: Using GPT-4 to annotate the severity of all phenotypic abnormalities within the human phenotype ontology
Source: Front Digit Health. 2026 May 21;8:1794934. doi: 10.3389/fdgth.2026.1794934 (PMC13233404; doi:10.3389/fdgth.2026.1794934)

$F_{\text{Welch}}(3, 1837.39) = 4448.55, p = 2.23\text{e-}308, \hat{\omega}_p^2 = 0.88, \text{CI}_{95\%} [0.87, 1.00], n_{\text{obs}} = 17,502$

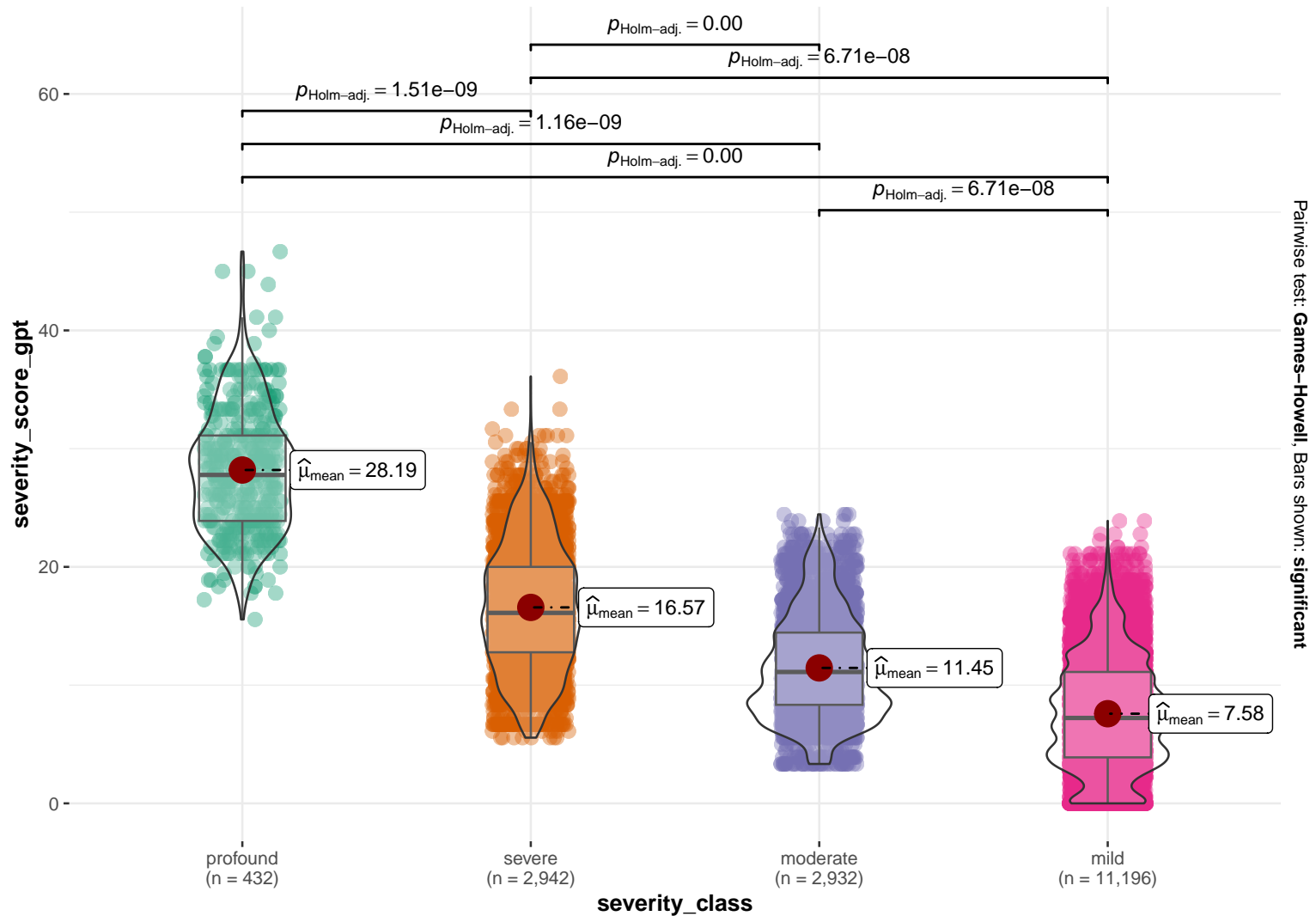

Supplement: Supplementary file 3 [file Image1.pdf]

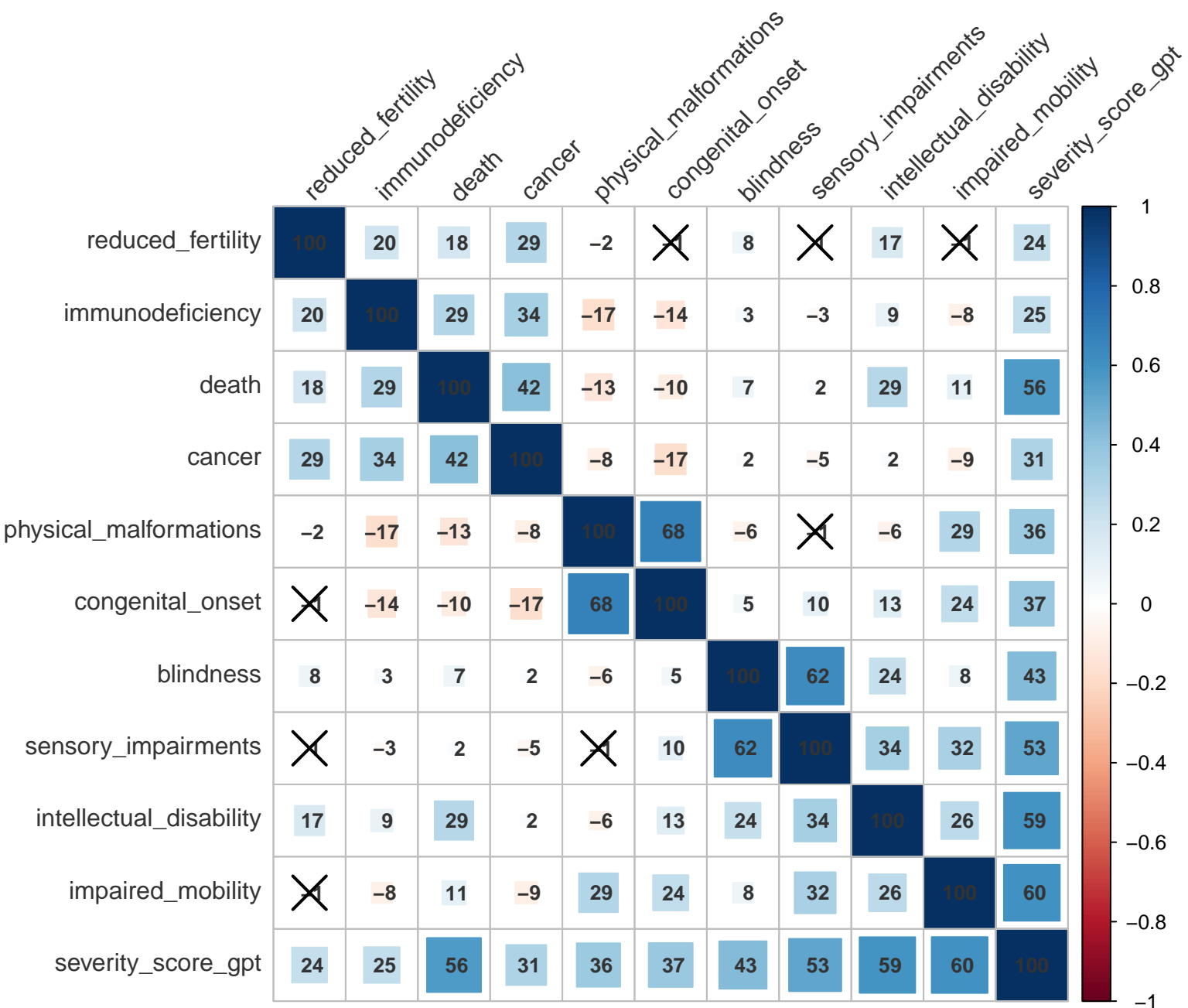

Supplement: Supplementary file 4 [file Image2.pdf]

Severity score

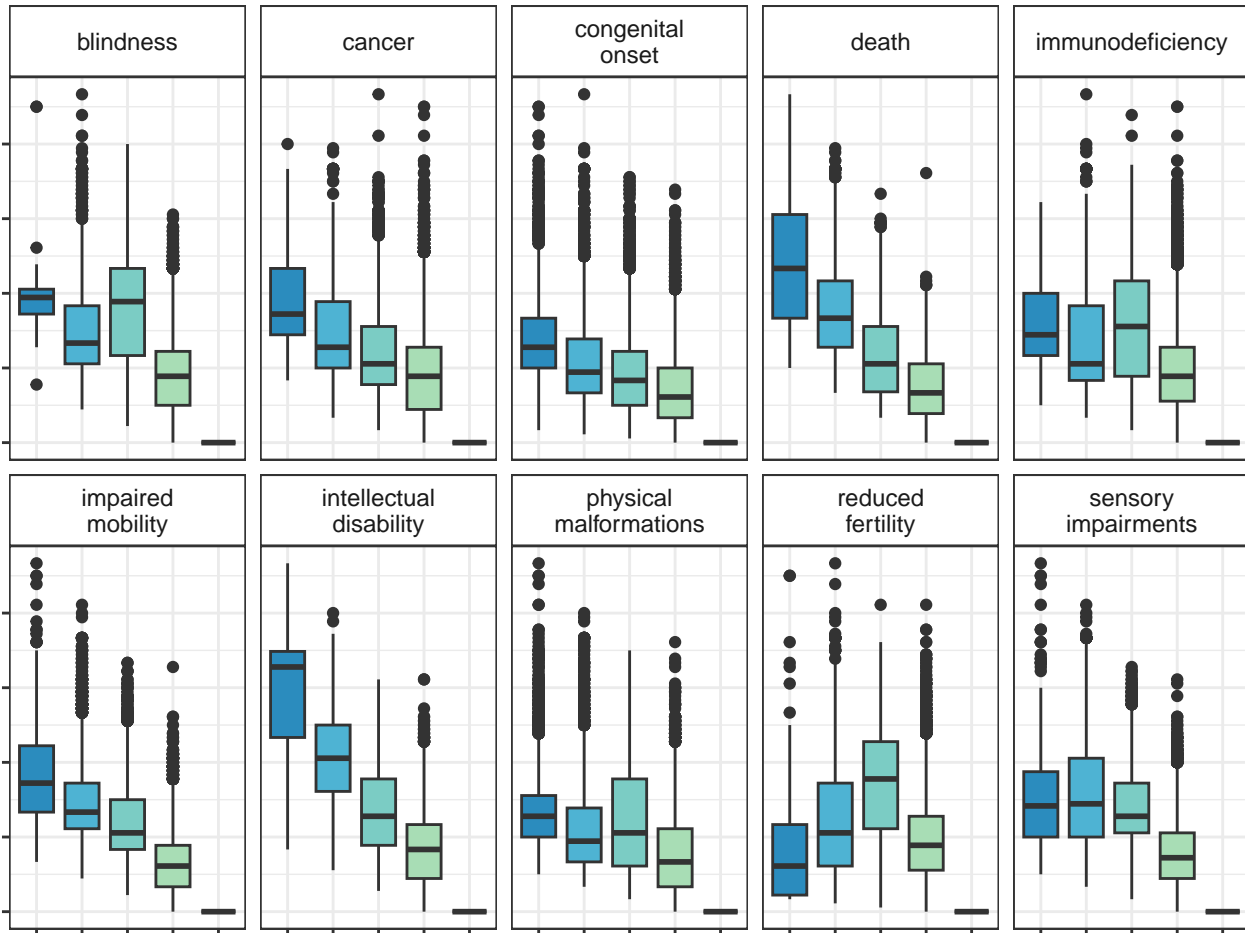

value

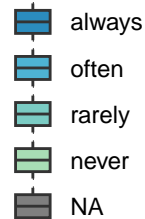

Supplement: Supplementary file 5 [file Image3.pdf]

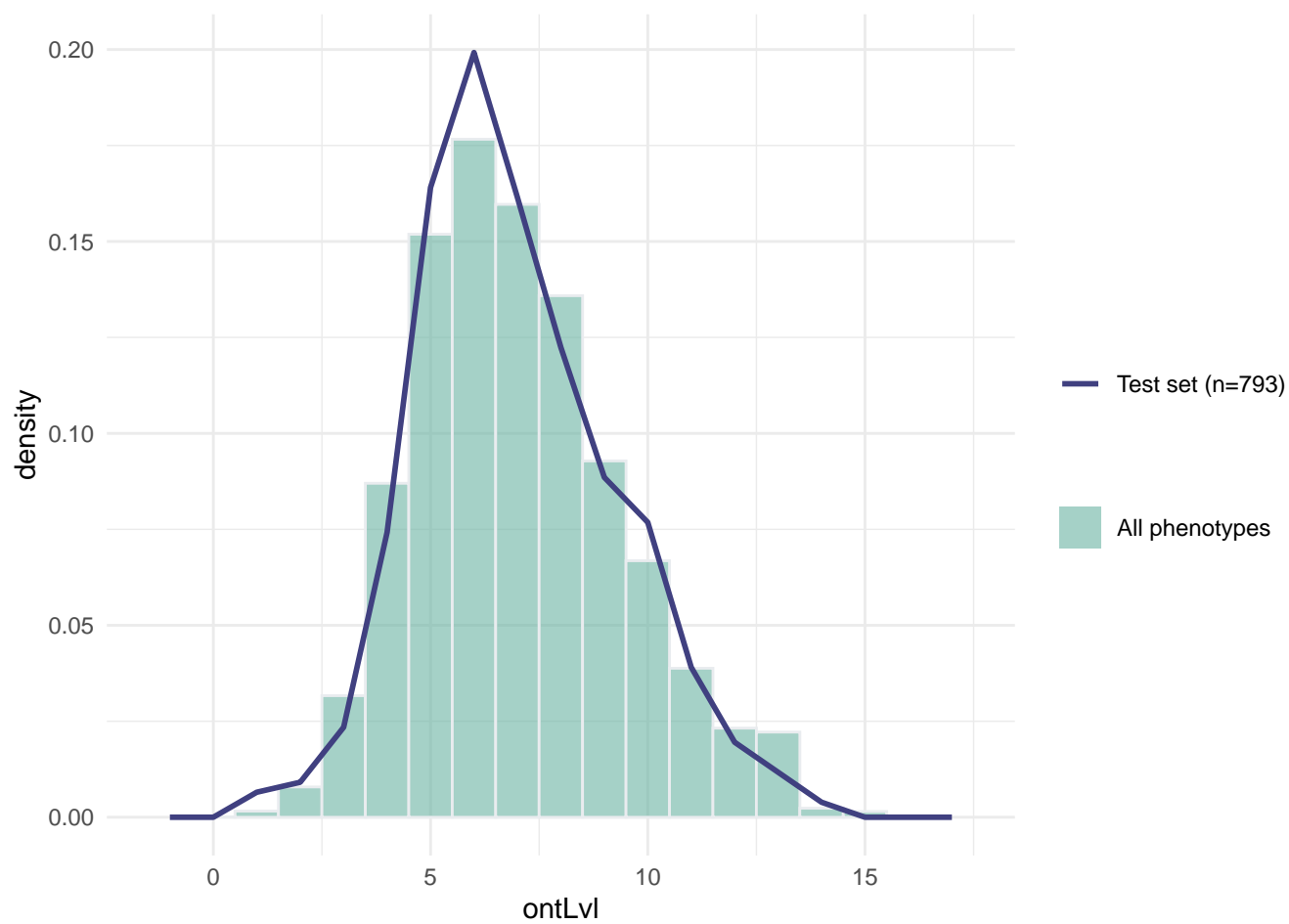

Supplement: Supplementary file 7 [file Image5.pdf]

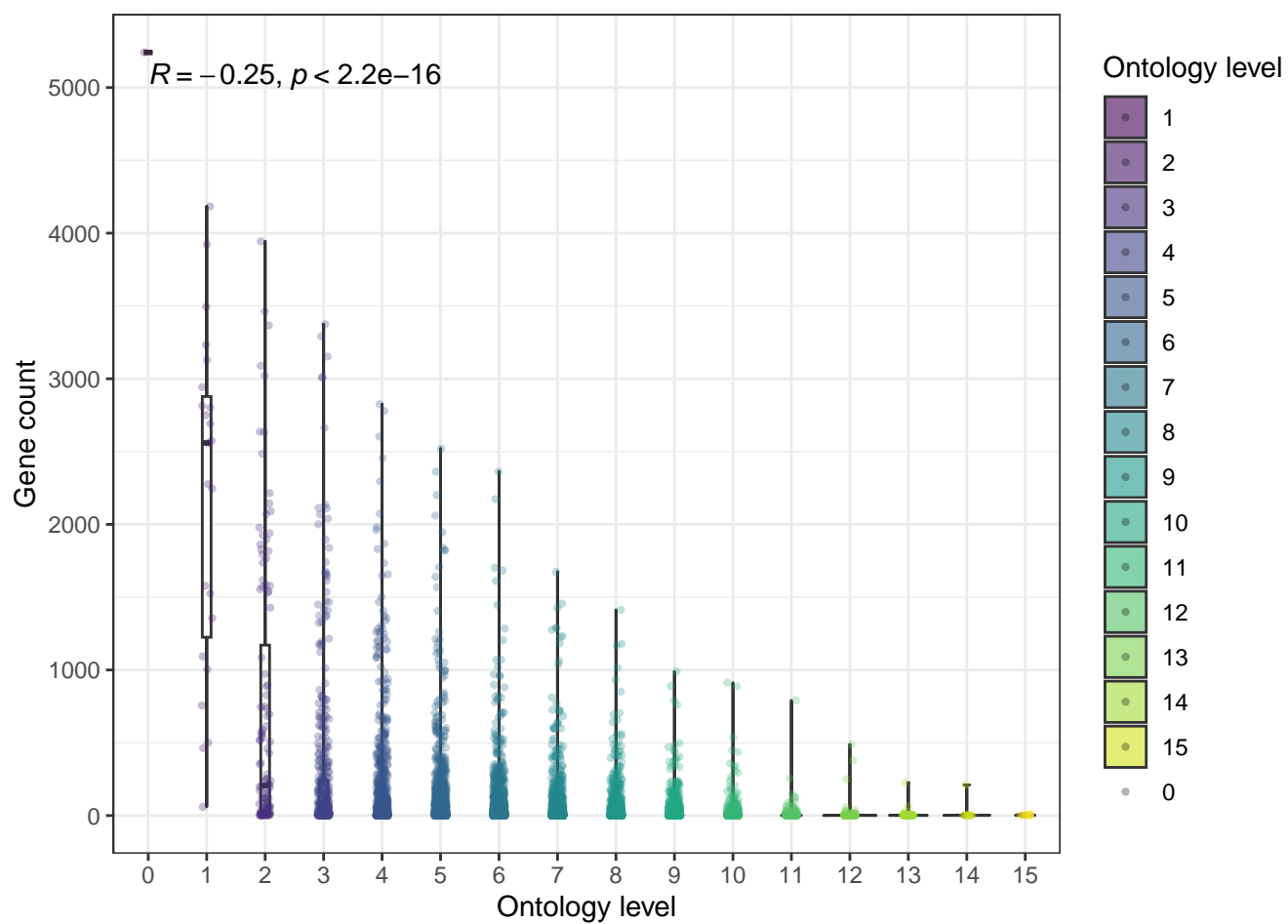

Supplement: Supplementary file 8 [file Image6.pdf]
