# Supplementary material for: Using GPT-4 to annotate the severity of all phenotypic abnormalities within the human phenotype ontology
Source: Front Digit Health. 2026 May 21;8:1794934. doi: 10.3389/fdgth.2026.1794934 (PMC13233404; doi:10.3389/fdgth.2026.1794934)
Supplement: Supplementary file 6 [file Image4.pdf]

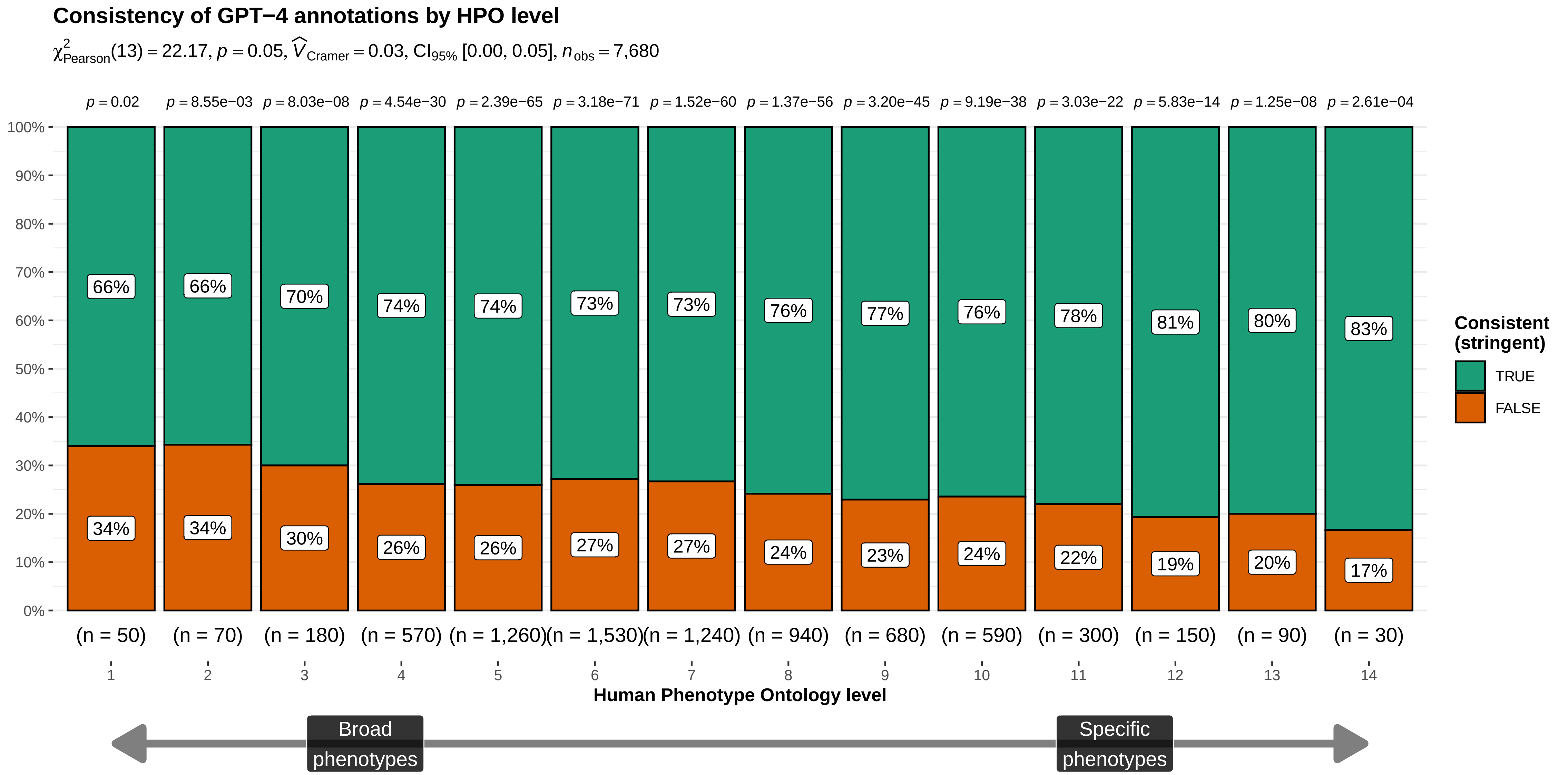

Consistency of GPT−4 annotations by HPO level
$\chi^2_{Pearson}(13) = 22.17, p = 0.05, \widehat{V}_{Cramer} = 0.03, CI_{95\%}$ [0.00, 0.05], $n_{obs} = 7,680$
$p = 0.02$
$p = 8.55e{-03}$
$p = 8.03e{-08}$
$p = 4.54e{-30}$
$p = 2.39e{-65}$
$p = 3.18e{-71}$
$p = 1.52e{-60}$
$p = 1.37e{-56}$
$p = 3.20e{-45}$
$p = 9.19e{-38}$
$p = 3.03e{-22}$
$p = 5.83e{-14}$
$p = 1.25e{-08}$
$p = 2.61e{-04}$
100%
90%
80%
70%
60%
50%
40%
30%
20%
10%
0%
66%
66%
70%
74%
74%
73%
73%
76%
77%
76%
78%
81%
80%
83%
34%
34%
30%
26%
26%
27%
27%
24%
23%
24%
22%
19%
20%
17%
(n = 50)
(n = 70)
(n = 180)
(n = 570)
(n = 1,260)
(n = 1,530)
(n = 1,240)
(n = 940)
(n = 680)
(n = 590)
(n = 300)
(n = 150)
(n = 90)
(n = 30)
1
2
3
4
5
6
7
8
9
10
11
12
13
14
Human Phenotype Ontology level
Consistent (stringent)
TRUE
FALSE
Broad phenotypes
Specific phenotypes
