# Supplementary material for: Using GPT-4 to annotate the severity of all phenotypic abnormalities within the human phenotype ontology
Source: Front Digit Health. 2026 May 21;8:1794934. doi: 10.3389/fdgth.2026.1794934 (PMC13233404; doi:10.3389/fdgth.2026.1794934)
Supplement: Supplementary file 9 [file Image7.pdf]

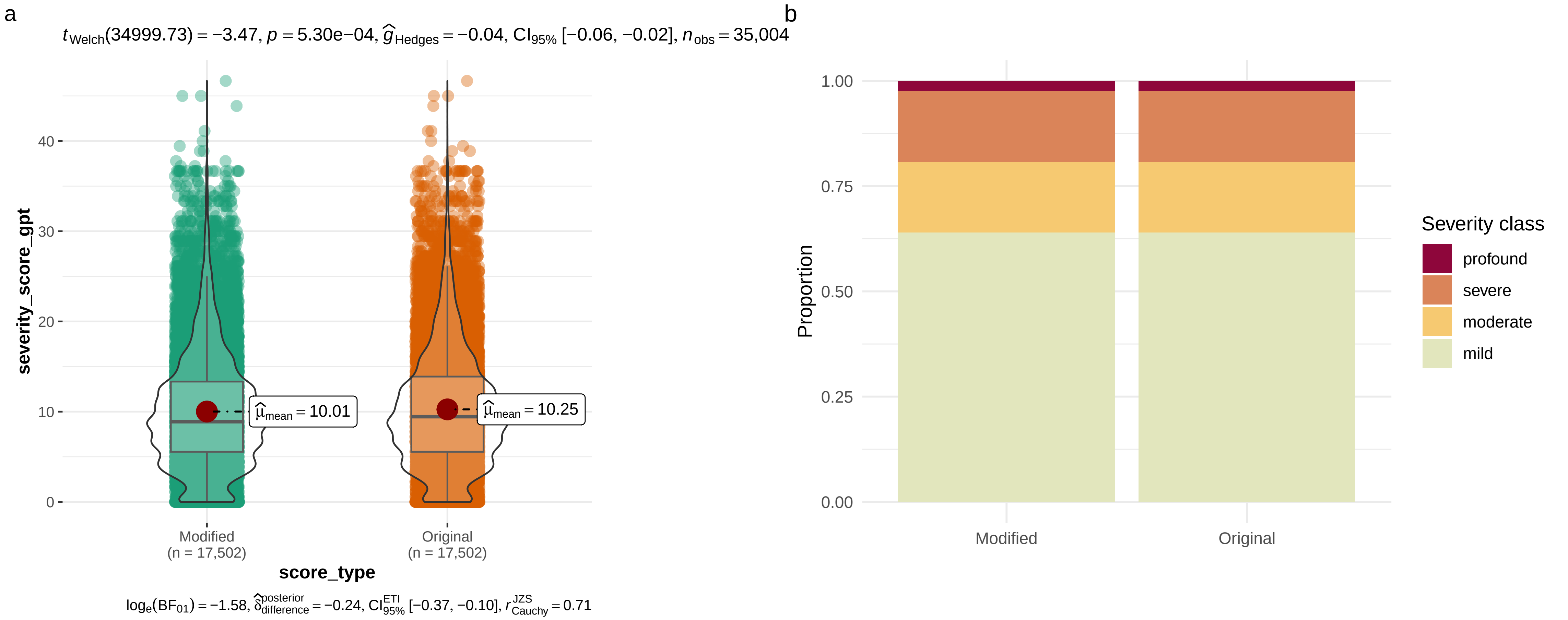
a
$t_{\text{Welch}}(34999.73) = -3.47$, $p = 5.30\text{e}{-04}$, $\widehat{g}_{\text{Hedges}} = -0.04$, $\text{CI}_{95\%}$ [−0.06, −0.02], $n_{\text{obs}} = 35{,}004$
severity_score_gpt
40
30
20
10
0
$\widehat{\mu}_{\text{mean}} = 10.01$
$\widehat{\mu}_{\text{mean}} = 10.25$
Modified
(n = 17,502)
Original
(n = 17,502)
score_type
$\log_e(\text{BF}_{01}) = -1.58$, $\widehat{\delta}_{\text{difference}}^{\text{posterior}} = -0.24$, $\text{CI}_{95\%}^{\text{ETI}}$ [−0.37, −0.10], $r_{\text{Cauchy}}^{\text{JZS}} = 0.71$
b
Proportion
1.00
0.75
0.50
0.25
0.00
Modified
Original
Severity class
profound
severe
moderate
mild
